# Selective and efficient H_2_ evolution upon NH_3_BH_3_ hydrolysis at subzero temperatures

**DOI:** 10.1016/j.isci.2023.108774

**Published:** 2023-12-22

**Authors:** Qing Zhang, Chen Fang, Yanlan Wang, Xiang Liu

**Affiliations:** 1 Engineering Research Center of Eco-Environment in Three Gorges Reservoir Region of Ministry of Education, College of Materials and Chemical Engineering, China Three Gorges University, Yichang, Hubei 443002, P.R. China; 2 Shandong Provincial Key Laboratory of Chemical Energy Storage and Nevel Cell Technology, Department of Chemistry and Chemical Engineering, Liaocheng University, Liaocheng 252059, China

**Keywords:** Electrochemistry, Applied sciences

## Abstract

In the winter months, the temperature in most of the Earth stays below 0°C; the average temperature in winter at the South Pole is about −60°C. Therefore, it is urgent to develop efficient catalytic systems for selective and efficient H_2_ evolution upon NH_3_BH_3_ hydrolysis at subzero temperatures. For solving the freezing issue of water at below 0°C, herein, we have employed a facile and surfactant-free approach to synthesize M-Pt/C nanocomposites (M = Pd, Rh, Ru, Ni, Cu, or Fe), by the alloying of commercial Pt/C with Pd, Rh, Ru, Cu, Ni, or Fe for selective and efficient H_2_ evolution upon NH_3_BH_3_ hydrolysis in saline solution at below 0°C, even at −15°C. In addition, NH_3_BH_3_ hydrolysis over Pd-Pt/C in the saturated NaCl solution is utilized not only for safe hydrogen production but also for its *in situ* hydrogenation reduction in organic chemistry, which could avoid using dangerous hydrogen cylinders.

## Highlights

• M-Pt/C nanocomposites were synthesized by alloying of commercial Pt/C with metals
• H_2_ evolution upon NH_3_BH_3_ hydrolysis in saline solution at below 0°C, even at −15°C
• Pd-Pt/C presented the superior TOF of 5357.1 h^−1^ and 1038 h^−1^ at 0°C and−15°C, respectively
• This study could solve the freezing problem of water in NH_3_BH_3_ hydrolysis at below 0°C

## Graphical abstract

**Figure:**
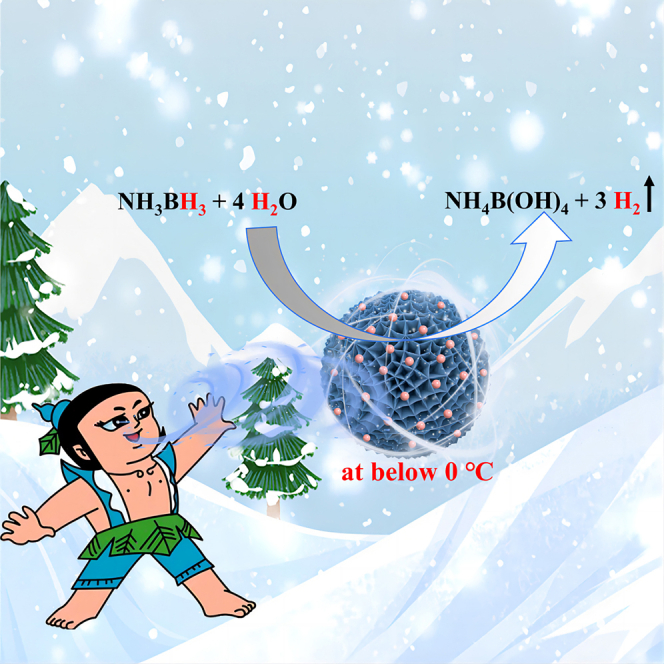

## Introduction

The ever-accelerating demand of the world’s total energy and greenhouse effect of CO_2_ from the combustion of fossil fuels have prompted a full-scale search for renewable and alternative energies (such as tide energy,^1^ sunlight,^2^ biomass,^3^ geothermal energy,^4^ wind,^5^ and H_2_^6^) in place of fossil fuel. Hydrogen (H_2_) is identified as the one of most cleanest energy alternatives to conventional fossil carbon-based fuels because of its highest density of energy,^7^^,^^8^^,^^9^^,^^10^ zero emission of carbon dioxide, and environmental benignity.^11^^,^^12^^,^^13^ The combustion of H_2_ only generates H_2_O, which could be applied to complete a hydrogen cycle *via* water splitting.^14^^,^^15^^,^^16^^,^^17^^,^^18^^,^^19^ However, the large-scale and practical application of hydrogen energy was severely limited by the safe storage, safe transport, and on-demand release of H_2_, because H_2_ is highly flammable, explosive, and difficult to be liquefied.^20^^,^^21^^,^^22^^,^^23^^,^^24^ Fortunately, H_2_ evolution from hydrogen storage materials (including methanol,^25^^,^^26^^,^^27^ formic acid,^28^^,^^29^^,^^30^ hydrazine hydrate,^31^^,^^32^ silicohydrides,^33^ sodium borohydride,^34^^,^^35^^,^^36^ and ammonia borane^37^^,^^38^^,^^39^^,^^40^) has been taken for as a safe and controllable approach to achieve an ideal model for hydrogen energy.^41^^,^^42^^,^^43^^,^^44^^,^^45^^,^^46^ Among them, H_2_ evolution upon NH_3_BH_3_ hydrolysis got a lot of attention in industry and academia by reason of its low molecular mass (31 g/mol), superior H_2_ concentration (19.6 wt %), non-toxicity, high stability, and water solubility.^47^^,^^48^^,^^49^^,^^50^^,^^51^

Equation 1 $${NH}_{3}{BH}_{3}+4\;H_{2}O\to {NH}_{4}B{\left(OH\right)}_{4}+3H_{2}↑$$

Indeed, a wide range of homogeneous and heterogeneous catalytic systems are designed and constructed for the selective and efficient H_2_ evolution upon NH_3_BH_3_ hydrolysis (Equation 1).^52^^,^^53^^,^^54^^,^^55^^,^^56^ Mounting evidences illustrated that the activation of the O-H bond of H_2_O was the rate-determining step in NH_3_BH_3_ hydrolysis.^57^^,^^58^^,^^59^^,^^60^^,^^61^ In the winter months, the temperature in most of the Earth stays below 0°C; e.g., the average temperature in winter at the South Pole is about −60°C.^62^ Therefore, it is of high significance to develop efficient catalytic systems for selective and efficient H_2_ evolution upon NH_3_BH_3_ hydrolysis at subzero temperatures. For solving the freezing problem of water at below 0°C, herein, we have employed a facile and surfactant-free approach to synthesize M-Pt/C nanocomposites (M = Pd, Rh, Ru, Ni, Cu, or Fe), by the alloying of commercial Pt/C with Pd, Rh, Ru, Cu, Ni, or Fe for selective and efficient H_2_ evolution upon NH_3_BH_3_ hydrolysis in saline solution at below 0°C, even at −15°C. Among them, Pt/C, as a heterogeneous and commercial catalyst, is widely applied in hydrogenation reactions because of its high capacity of Pt for H_2_ activation. Then, we scrutinized the kinetics of M-Pt/C nanomaterials for NH_3_BH_3_ hydrolysis in saline solution, the mechanistic aspects of NH_3_BH_3_ hydrolysis using kinetic isotope effect (KIE) experiment, and tandem reaction and gas chromatography (GC) analysis.

## Results and discussion

### Characterization of M-Pt/C nanomaterials

**Scheme 1:**
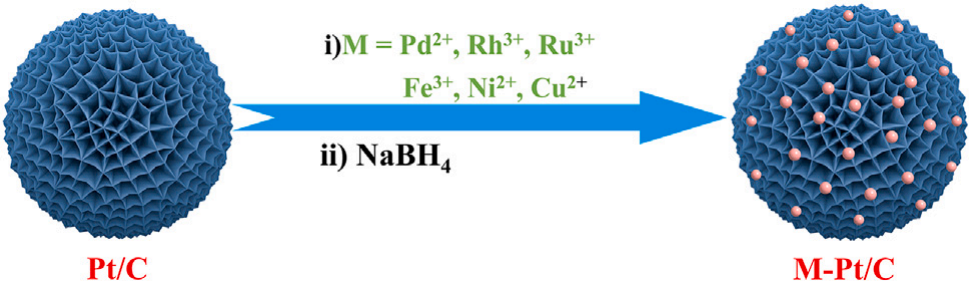
The synthesis of M-Pt/C

**Figure 1:**
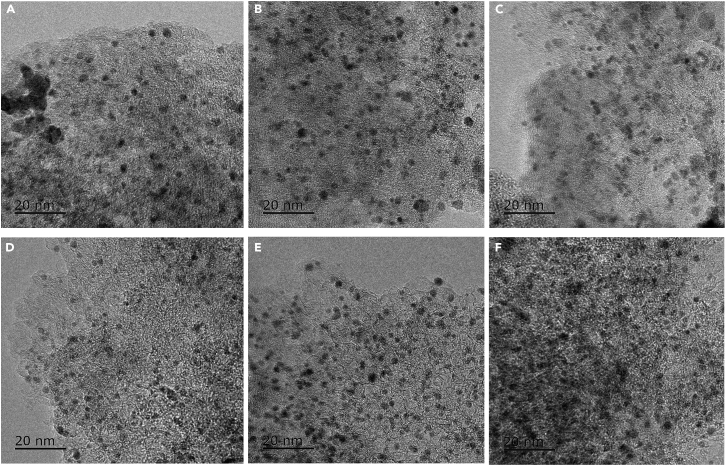
The morphology of M-Pt/C (A–F) TEM of (A) Pd-Pt/C, (B) Rh-Pt/C, (C) Ru-Pt/C, (D) Ni-Pt/C, (E) Cu-Pt/C, and (F) Fe-Pt/C.

**Figure 2:**
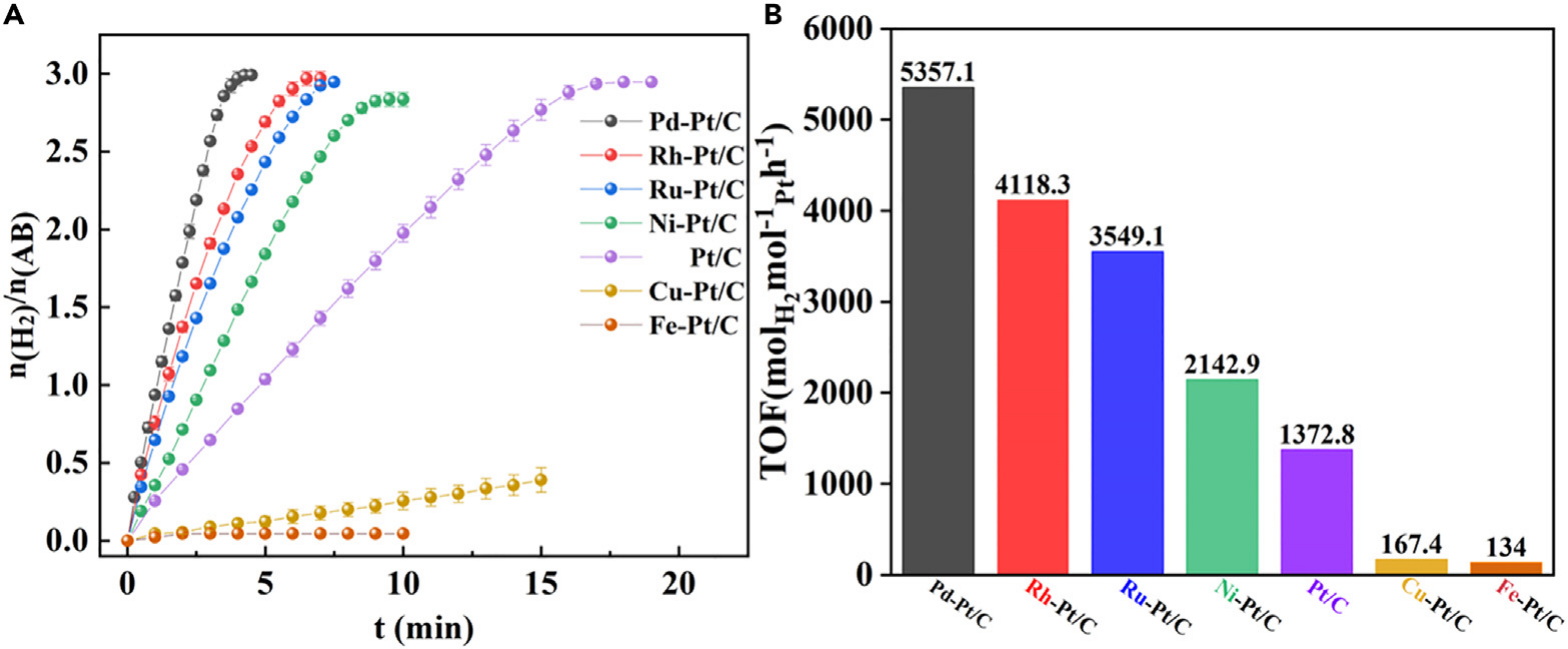
The catalytic activities of M-Pt/C in NH_3_BH_3_ hydrolysis (A) Comparison of NH_3_BH_3_ hydrolysis by 1 mol % M-Pt/C nanomaterials in saline solution at 0°C. (B) their corresponding TOF. TOF = n(H_2_)/n(Pt)·t.

First, M-Pt/C nanomaterials (Pd-Pt/C, Rh-Pt/C, Ru-Pt/C, Ni-Pt/C, Cu-Pt/C, and Fe-Pt/C) were successfully synthesized, *via* a fast reduction of NaBH_4_ at 30°C, by the alloying of commercial Pt/C and Pd, Rh, Ru, Ni, Cu, or Fe with the mole rate of 1.0 : 1.0 (Scheme 1). As illustrated in Figure S1, we found the mean size of commercial Pt/C was 1.72 nm (Figure S2). Next, we found the size of M-Pt/C was slightly larger than that of Pt/C, suggesting that Pd, Rh, Ru, Ni, Cu, or Fe were alloyed onto Pt/C. As recorded in Figure 1, the mean size of M-Pt/C was about 2 nm. The size of Pd-Pt/C (Figure 1A), Rh-Pt/C (Figure 1B), Ru-Pt/C (Figure 1C), Ni-Pt/C (Figure 1D), Cu-Pt/C (Figure 1E), and Fe-Pt/C (Figure 1F) was 1.90 nm (Figure S3), 1.90 nm (Figure S4), 1.90 nm (Figure S5), 2.13 nm (Figure S6), 1.97 nm (Figure S7), and 1.90 nm (Figure S8), respectively. For comparison, the as-synthesized Pd-Pt/C, Rh-Pt/C, Ru-Pt/C, Ni-Pt/C, Cu-Pt/C, Fe-Pt/C, and commercialized Pt/C were measured for H_2_ evolution upon NH_3_BH_3_ hydrolysis in saturated NaCl solution at 0°C (Figure 2A). The result exhibited that the catalytic performance order of M-Pt/C nanomaterials in H_2_ evolution upon NH_3_BH_3_ hydrolysis in saturated NaCl solution at 0°C is as follows: Pd-Pt/C (5357.1 h^−1^) > Rh-Pt/C (4118.3 h^−1^) > Ru-Pt/C (3549.1 h^−1^) > Ni-Pt/C (2142.9 h^−1^) > Pt/C (1372.8 h^−1^) > Cu-Pt/C (167.4 h^−1^) > Fe-Pt/C (134 h^−1^). Hence, Pd-Pt/C, with the turnover frequency (TOF) of 5357.1 mol(H_2_)·mol_Pt_^−1^·h^−1^ (2678.5 mol(H_2_)·mol_PtPd_^−1^·h^−1^), was chosen for further kinetic research, KIE experiment, and tandem reaction.

**Figure 3:**
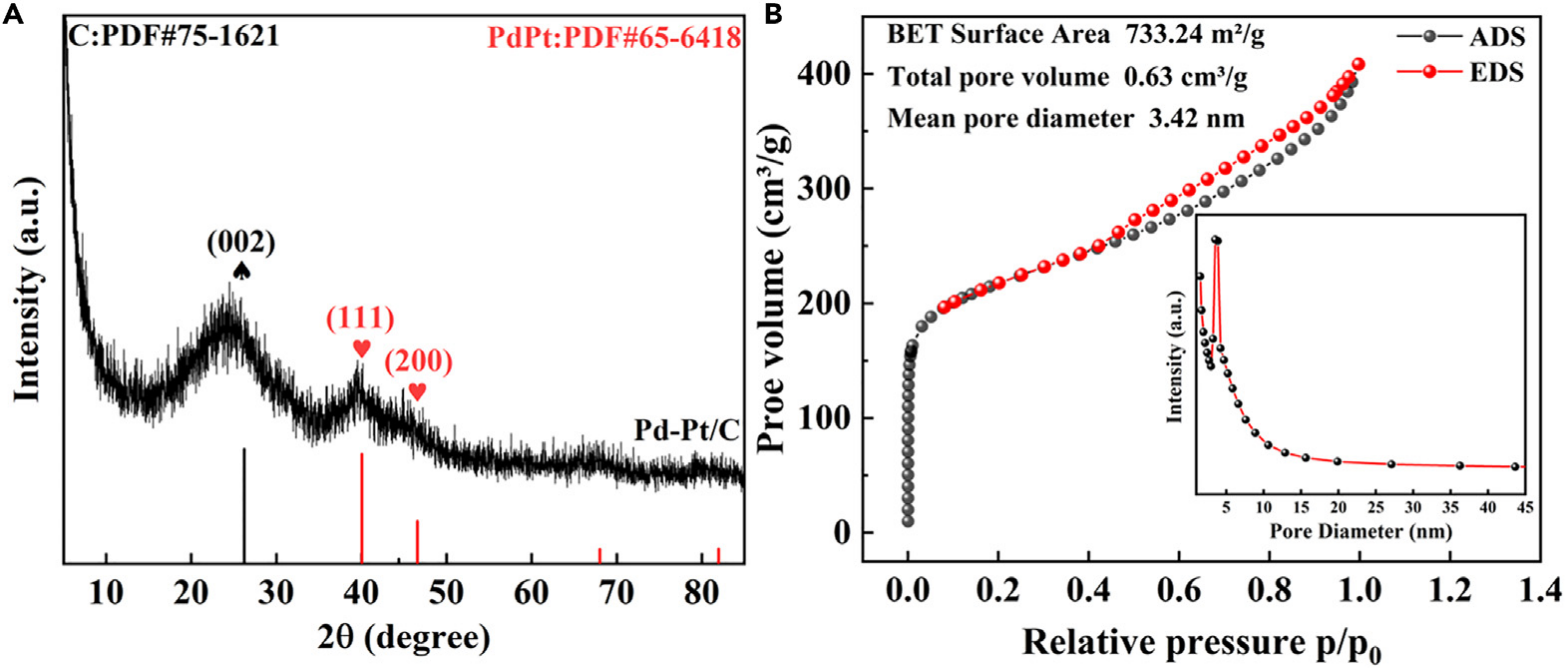
The physical characterization of Pd-Pt/C (A and B) (A) XRD and (B) BET of Pd-Pt/C.

**Figure 4:**
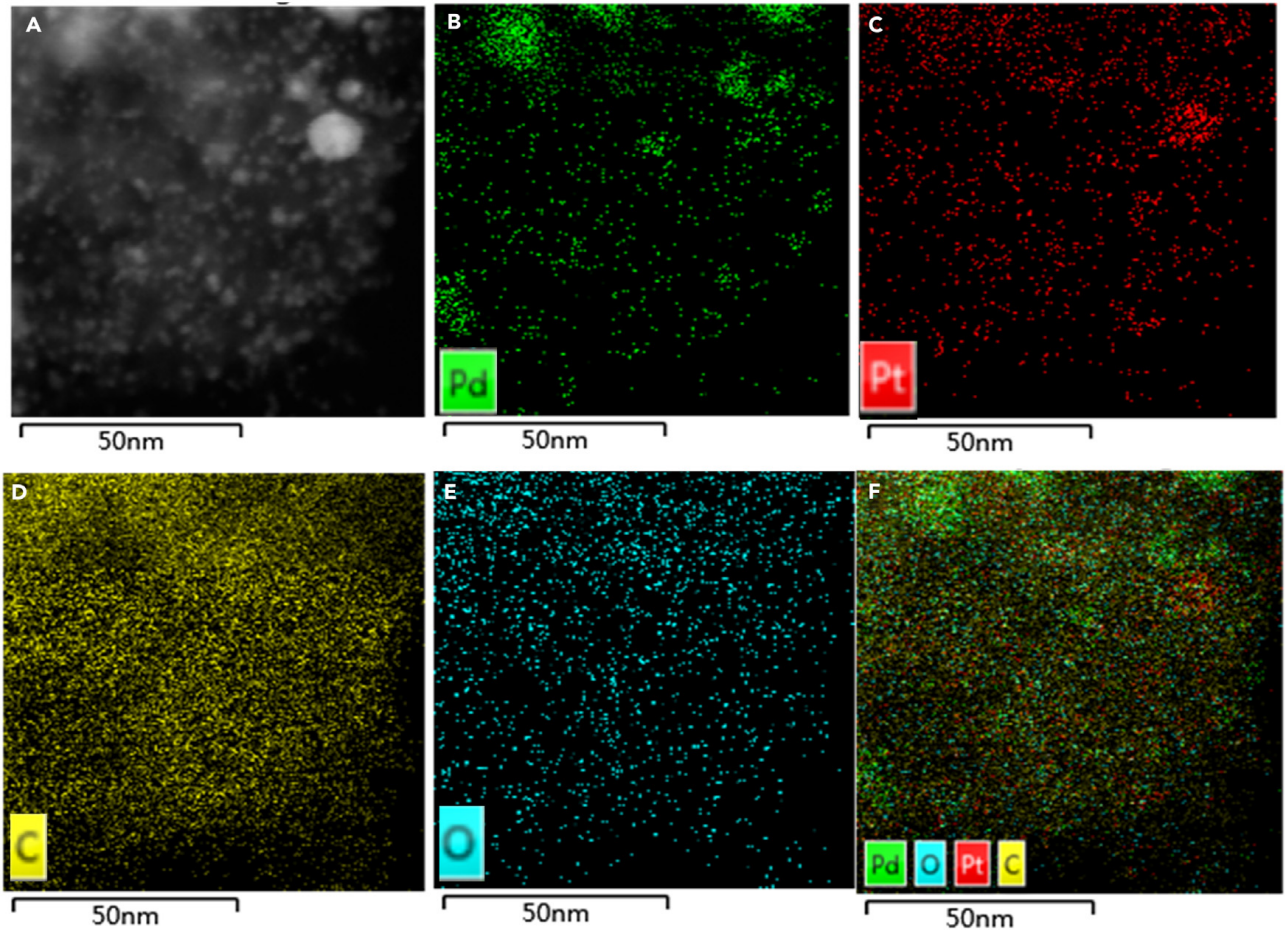
The high resolution morphology of Pd-Pt/C (A–F) (A) HADDF-STEM, (B) Pd, (C) Pt, (D) C, (E) O, and (F) combined (Pd, O, Pt, and C) EDX compositional mapping of Pd-Pt/C.

**Figure 5:**
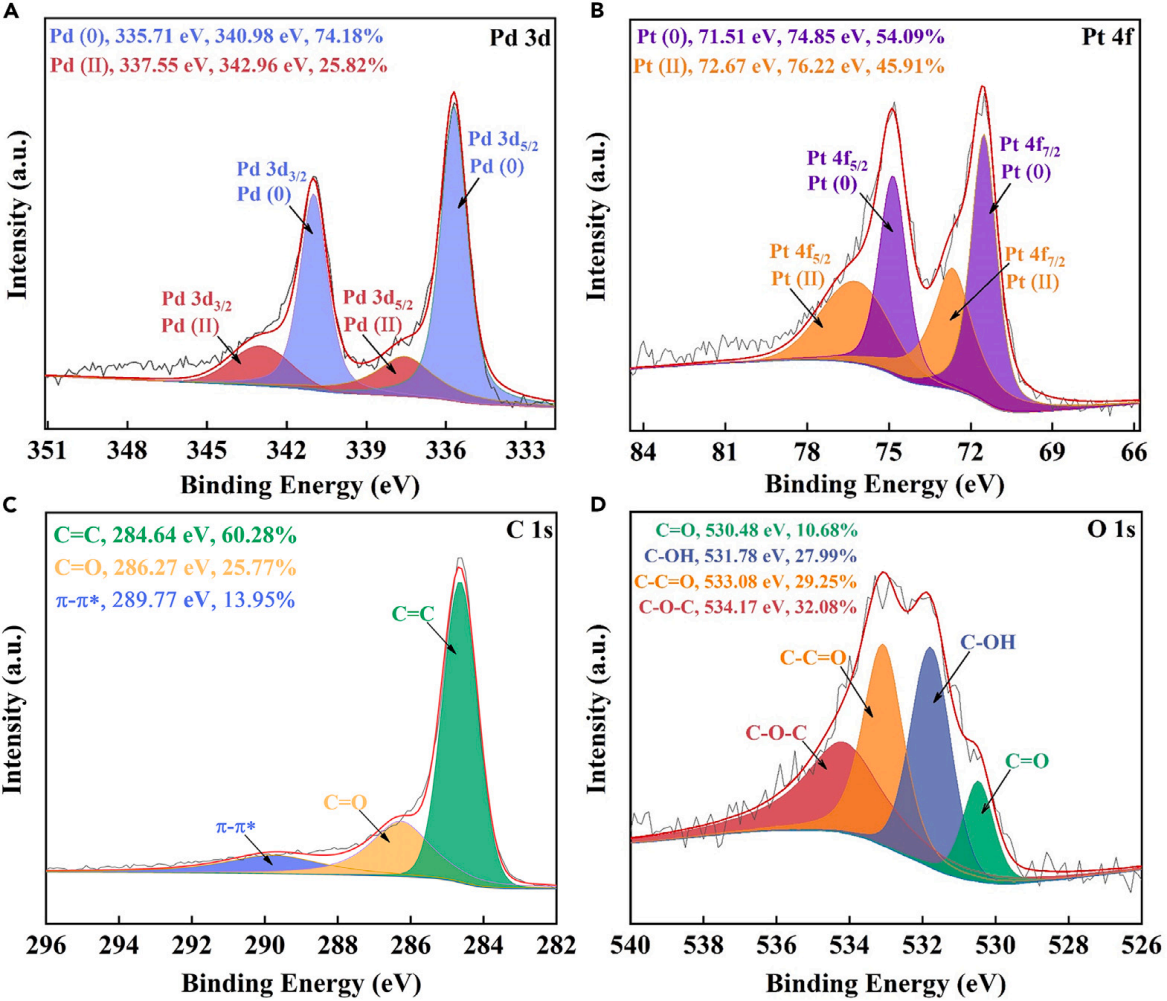
XPS of Pd-Pt/C (A–D) (A) Pd 3d, (B) Pt 4f, (C) C 1s, and (D) O 1s XPS of Pd-Pt/C.

Three characteristic peaks at 26°, 40°, and 47° were attributed to plane lattice of C (002), PdPt (111), and PdPt (200), respectively (No. 75-1621 and 65-6418).^63^^,^^64^ Nitrogen adsorption-desorption analysis (BET) of Pd-Pt/C nanocomposite exhibited that its pore volume, pore diameter, and specific surface area were 3.42 nm, 0.63 cm^3^/g, and 733.24 m^2^/g, respectively (Figure 3B). In order to investigate detailed compositions of Pd-Pt/C, its Energy Dispersive X-ray Detector (EDX) was recorded. As depicted in Figures 4A–4E, the distributions of Pt, C, and Pd proved the strong adsorption of Pd to Pt/C surface. In particular, both Pd and Pt were mostly located at the same places (Figures 4B and 4C), further confirming the existence of PdPt alloy structure. In Figure 4F, the whole surface of Pd-Pt/C was made up of Pd, Pt, O, and C. In the high resolution transmission electron microscopy (HRTEM), the crystal lattice spacing of 0.22 nm, which was attributed to PdPt (111), was clearly recorded in Figure S9, verifying the generation of PdPt alloy in Pd-Pt/C. Moreover, the chemical valence states of Pd-Pt/C were further studied by X-ray photoelectron spectroscopy (XPS). As presented in Figure 5A, the two characteristic peaks of Pd 3d_3/2_ at 342.96 eV and Pd 3d_5/2_ at 337.55 eV were attributed to Pd^II^ (25.82%), whereas the other pair peaks of Pd 3d_3/2_ (340.98 eV) and Pd 3d_5/2_ (335.71 eV) were ascribed to Pd^0^ (74.18%). As shown in Figure 5B, in similar, the two peaks of Pt 4f_5/2_ located at 76.22 eV and Pt 4f_7/2_ at 72.67 eV were attributed to Pt^II^ (45.91%), whereas the other pair peaks of Pt 4f_5/2_ (74.85 eV) and Pt 4f_7/2_ (71.51 eV) were ascribed to Pt^0^ (54.09%). In the C 1s spectrum (Figure 5C), three characteristic peaks of 284.64 eV, 286.27 eV, and 289.77 eV were defined to C=C (60.28%), C=O (25.77%), and π-π∗ (13.95%), respectively. As shown in Figure 5D, O 1s spectrum had been divided into 4 characteristic peaks of 530.48 eV, 531.78 eV, 533.08 eV, and 534.17 eV, being assigned to C=O (10.68%), C-OH (27.99%), C-C=O (29.25%), and C-*O*-C (32.08%), respectively. Compared to the Pt 4f of Pt/C, an obvious right shift was recorded in the Pt 4f of Pd-Pt/C (Figure S10), suggesting the electron was transferred from Pd atom into the surface of Pt atom. This led to rich electron cloud density of Pd atom surface, which was favorable for catalysis.^65^

### Kinetic study

**Figure 6:**
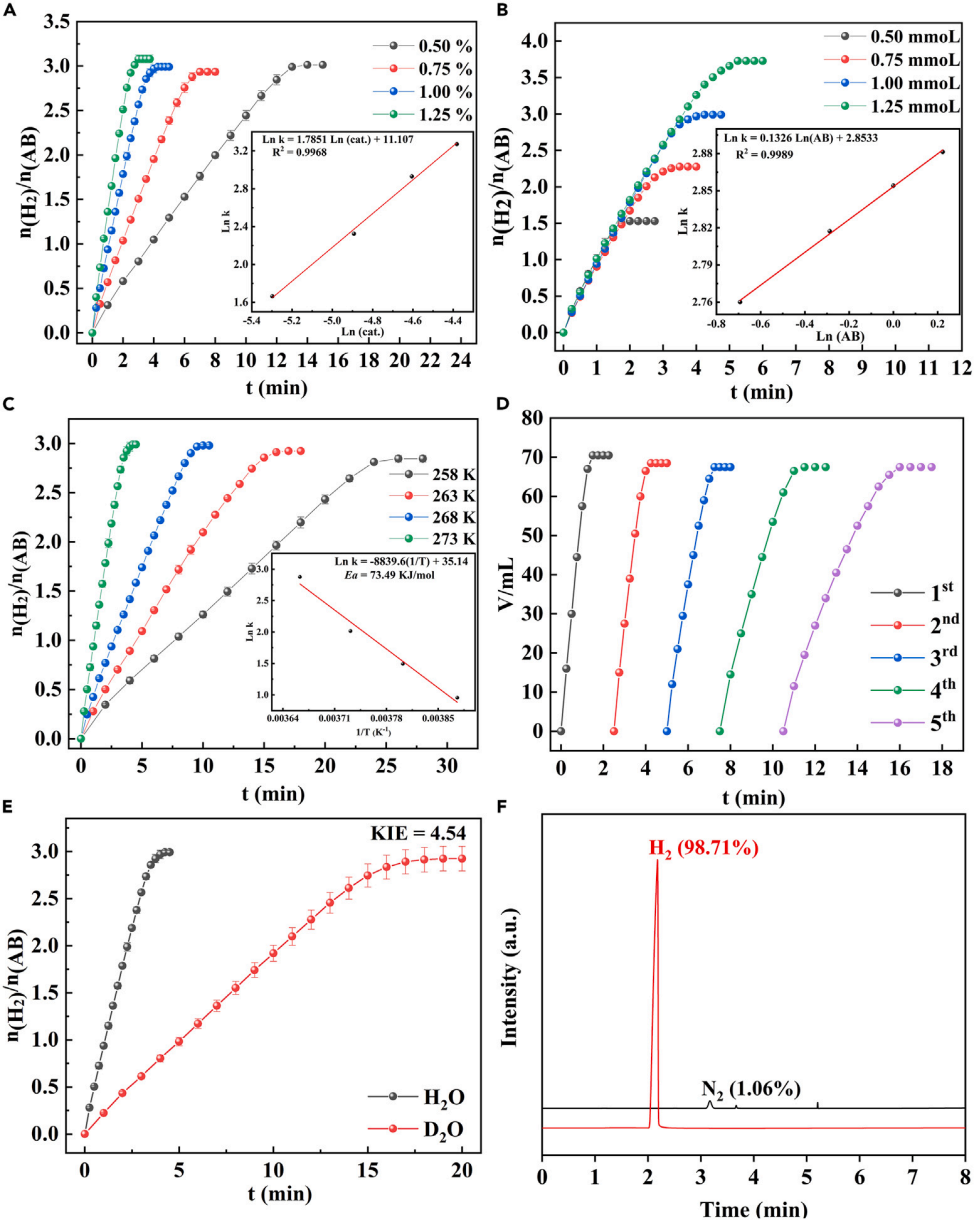
Kinetic study of Pd-Pt/C in NH_3_BH_3_ hydrolysis (A and B) Plots of the volume of produced H_2_ versus time for (A) NH_3_BH_3_ hydrolysis with various dosages of Pd-Pt/C, (B) different amounts of NH_3_BH_3_ in the saturated NaCl solution at 0°C. (C) NH_3_BH_3_ hydrolysis catalyzed by 1 mol % Pd-Pt/C in the saturated NaCl solution at various reaction temperatures; (D) Stability of Pd-Pt/C in NH_3_BH_3_ hydrolysis in the saturated NaCl solution at 0°C. (E) NH_3_BH_3_ hydrolysis catalyzed by 1 mol % Pd-Pt/C with H_2_O and D_2_O in the saturated NaCl solution at 0°C, respectively. (F) GC spectra of releasing gas from NH_3_BH_3_ hydrolysis in the saturated NaCl solution at 0°C.

The kinetics study of NH_3_BH_3_ hydrolysis in the saturated NaCl solution (including Pd-Pt/C dosages, NH_3_BH_3_ concentration, and hydrolysis temperature) had been investigated for further industrialization. The NH_3_BH_3_ hydrolysis was conducted with various dosages of Pd-Pt/C (0.5–1.25 mol %). As illustrated in Figure 6A, the H_2_ generation rate increased gradually with the increase of Pd-Pt/C dosages. The slope was 1.79 in the logarithmic scale, illustrating a good linear relationship between them. However, the H_2_ generation rate was independent of the concentration of NH_3_BH_3_ (Figure 6B). It is distinct that NH_3_BH_3_ hydrolysis was the zero-order kinetic in the concentration of NH_3_BH_3_. More importantly, H_2_ generation upon NH_3_BH_3_ hydrolysis over Pd-Pt/C in the saturated NaCl solution was carried out at different reaction temperatures from −15°C to 0°C (Figure 6C). It is clear that H_2_ was successfully generated from NH_3_BH_3_ hydrolysis over Pd-Pt/C in the saturated NaCl solution at below 0°C, even at −15°C, which absolutely solved the freezing problem of water at below 0°C. According to Arrhenius law, the *E*_*a*_ of H_2_ generation upon NH_3_BH_3_ hydrolysis over Pd-Pt/C in saturated NaCl solution was 73.49 kJ/mol.

### Stability of Pd-Pt/C

The stability of catalyst played a critical role in the H_2_ generation upon NH_3_BH_3_ hydrolysis for the potential industrialization. Cyclic experiment was conducted with 1 mmol of NH_3_BH_3_ and 2 mol % of Pd-Pt/C in the saturated NaCl solution at 0°C. First, Pd-Pt/C catalyst had been separated and reused by the centrifugalization, when NH_3_BH_3_ hydrolysis was finished. Then another NH_3_BH_3_ and the saturated NaCl solution was put into the reaction for the next H_2_ evolution. As illustrated in Figure 6D, the result exhibited that Pd-Pt/C catalyst was successfully recycled at least 5 times in H_2_ generation upon NH_3_BH_3_ hydrolysis in the saturated NaCl solution at 0°C, with just slight loss of catalytic efficiency. In addition, the 5^th^ reused Pd-Pt/C was further measured by TEM. As described in Figure S11, the mean size of 5^th^ recycled Pd-Pt/C increased from 1.72 nm to 2.10 nm (Figure S12), and no significant change in the morphology and crystal structure was detected. This confirmed that Pd-Pt/C is a real heterogeneous nano-catalyst for H_2_ evolution upon NH_3_BH_3_ hydrolysis.

### Mechanism study

**Scheme 2:**
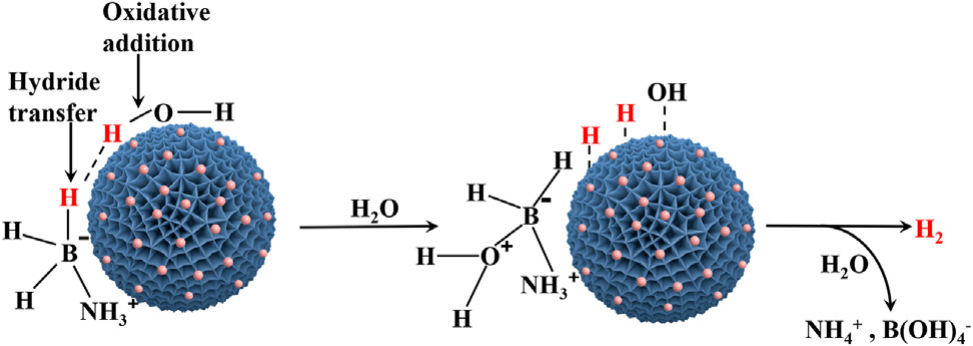
The suggested mechanism of NH_3_BH_3_ hydrolysis over Pd-Pt/C

For revealing the mechanism of NH_3_BH_3_ hydrolysis, H_2_ generation upon NH_3_BH_3_ hydrolysis over Pd-Pt/C in the saturated NaCl solution was performed in D_2_O and H_2_O. As demonstrated in Figure 6E, a fairly large KIE of 4.54 was acquired, suggesting that the destruction of O-H bond of H_2_O was the rate-determining step for H_2_ generation upon NH_3_BH_3_ hydrolysis over Pd-Pt/C in the saturated NaCl solution.^65^ As shown in Figure 6F, the gas generated from NH_3_BH_3_ hydrolysis over Pd-Pt/C in the saturated NaCl solution was also identified to be only H_2_ by Gas Chromatography (GC), further verifying that H_2_ generation upon NH_3_BH_3_ hydrolysis over Pd-Pt/C could be implemented for fuel cell at below 0°C.^66^ Based on KIE, GC, and relevant literature,^67^^,^^68^^,^^69^ a feasible mechanism of NH_3_BH_3_ hydrolysis over Pd-Pt/C was presented in Scheme 2. First, NH_3_BH_3_ molecules and H_2_O molecules were adsorbed and anchored at the surface of Pd-Pt/C. The hydridic H-B bond of NH_3_BH_3_ reacted with acidic H-O bond of H_2_O to invertibly afford the hydrogen-bridge of [HO-H … H-BH_2_NH_3_] at Pd-Pt/C surface. Then, a fairly large KIE of 4.54 acquired with D_2_O illustrated that the destruction of O-H bond of H_2_O was the rate-determining step for H_2_ generation upon NH_3_BH_3_ hydrolysis over Pd-Pt/C in the saturated NaCl solution. Finally, H_2_ gas was obtained by reductive elimination of metal hydrides.^70^

### Hydrogenation reduction

**Scheme 3:**
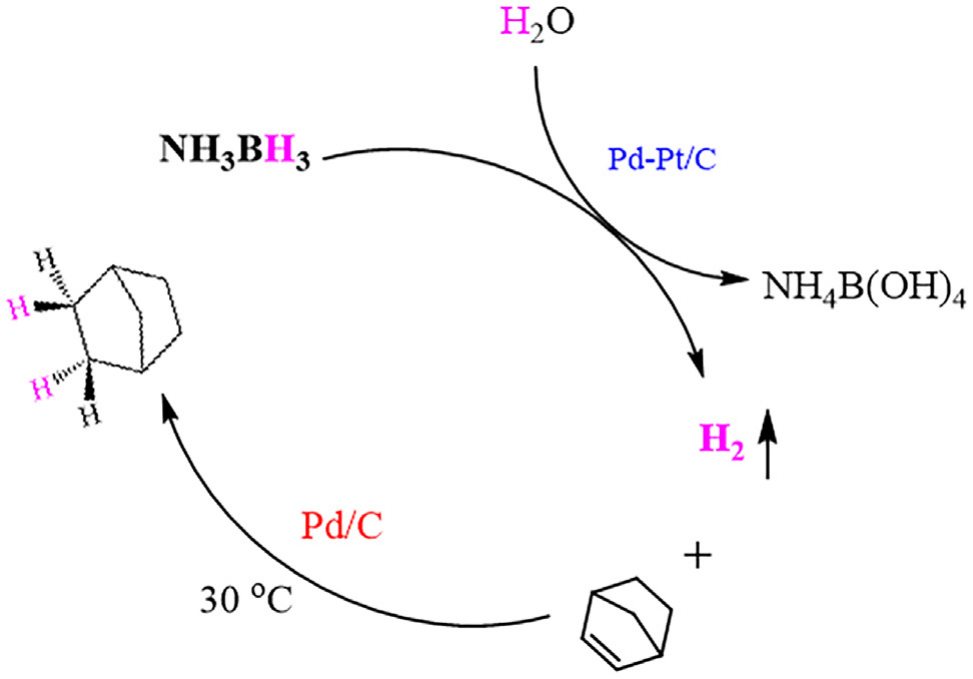
Hydrogenation reduction of norbornene

NH_3_BH_3_ hydrolysis over Pd-Pt/C in the saturated NaCl solution is utilized not only for safe H_2_ production at below 0°C but also for its *in situ* hydrogenation reduction in organic chemistry.^71^ The hydrogenation reduction of norbornene was carried out in a dual-chamber reactor with high gas tightness (Figure S13). The left chamber was utilized for NH_3_BH_3_ hydrolysis, and the right chamber was utilized for hydrogenation reduction of norbornene with the *in situ* released H_2_ from left chamber. As presented in Scheme 3, ^1^H-nuclear magnetic resonance (NMR) exhibited the target product was given in >99% yield (Figure S14), illustrating that H_2_ generation upon NH_3_BH_3_ hydrolysis over Pd-Pt/C in saturated NaCl solution at below 0°C was successfully utilized for *in situ* hydrogenation reduction in organic chemistry.^72^

### Conclusion

In summary, a facile and surfactant-free approach had been employed to construct M-Pt/C nanocomposites (M = Pd, Rh, Ru, Ni, Cu, or Fe), by the alloying of commercial Pt/C with Pd, Rh, Ru, Cu, Ni, or Fe for selective and efficient H_2_ evolution upon NH_3_BH_3_ hydrolysis in the saturated NaCl solution at below 0°C, even at −15°C. The result exhibited that the catalytic activity’s order of M-Pt/C in H_2_ evolution upon NH_3_BH_3_ hydrolysis in saturated NaCl solution at 0°C is as follows: Pd-Pt/C > Rh-Pt/C > Ru-Pt/C > Ni-Pt/C > Pt/C > Cu-Pt/C > Fe-Pt/C. It is clear that H_2_ was successfully generated from NH_3_BH_3_ hydrolysis over Pd-Pt/C in saturated NaCl solution at below 0°C, even at −15°C, which absolutely solved the freezing problem in the H_2_ evolution upon NH_3_BH_3_ hydrolysis at below 0°C. In addition, NH_3_BH_3_ hydrolysis over Pd-Pt/C is utilized not only for safe H_2_ production at below 0°C but also for its *in situ* hydrogenation reduction in organic chemistry, which could avoid using dangerous hydrogen cylinders.

### Limitations of the study

In this work, we focused on the selective and efficient H_2_ evolution upon NH_3_BH_3_ hydrolysis in the saturated NaCl solution at below 0°C. We speculate that the destruction of O-H bond of H_2_O was the rate-determining step for H_2_ generation upon NH_3_BH_3_ hydrolysis over Pd-Pt/C in the saturated NaCl solution. Thus, future research on the mechanistic investigations will be studied by density functional theory calculation.

## STAR★Methods

### Key resources table

**Table:** 

REAGENT or RESOURCE	SOURCE	IDENTIFIER
**Chemicals, peptides, and recombinant proteins**		

Platinum on carbon (Pt/C, 99.99%)	Aladdin Reagent Co., Ltd	7440-06-4
Sodium borohydride (NaBH_4_, 97%)	Shanghai Lingfeng Chemical Reagent Co., Ltd.	16940-66-2
Ruthenium trichloride (RuCl_3_, 99%)	Wuhan GeAo Reagent Co., Ltd	14898-67-0
Deuterium oxide (D_2_O, 99.9% atom % D)	Adamas-beta Reagent Co., Ltd	7789-20-0
Potassium chloropalladite (K_2_PdCl_4_, 99.99%)	Bidepharm Co., Ltd.	10025-98-6
Borane Ammonia complex (NH_3_BH_3_, 99.9%)	Bidepharm Co., Ltd.	13774-81-7
Rhodium nitrate solution (Rh(NO_3_)_2_, 99%)	Shanghai Macklin Biochemical Co., Ltd.	10139-58-9
Nickel chloride hexahydrate (NiCl_2_·6H_2_O, 99%)	Shanghai Macklin Biochemical Co., Ltd.	7791-20-0
Iron(Ⅲ) nitrate nonahydrate (Fe(NO_3_)_2_·9H_2_O, 99.9%)	Shanghai Macklin Biochemical Co., Ltd.	7758-98-7
Copper nitrate hydrate (Cu(NO_3_)_2_·xH_2_O, 99%)	Shanghai Macklin Biochemical Co., Ltd.	13778-31-9
Bicyclo[2.2.1]hept-2-ene (C_7_H_10_, 99%)	Shanghai Macklin Biochemical Co., Ltd.	498-66-8
Anhydrous methanol (CH_3_OH, 97%)	Sinopharm Chemical Reagent Co., Ltd.	67-56-1

#### Lead contact

Further information and requests for resources and reagents should be directed to and will be fulfilled by the Lead Contact, Xiang Liu (e-mail: xiang.liu@ctgu.edu.cn).

#### Materials availability

This study did not generate new unique reagents. Requests for resources and reagents should be directed to and will be fulfilled by the lead contact, Xiang Liu (e-mail: xiang.liu@ctgu.edu.cn.)

#### Data and code availability

• Any additional information required to reanalyze the data reported in this paper is available from the lead contact upon request.

### Experimental model and study participant details

This paper does not report any bio-experiments and any additional information required to reanalyse the data reported in this paper is available from the lead contact upon.

### Method details

#### The synthesis of Pd-Pt/C

First, 39 mg of dried Pt/C (0.01 mmol Pt), 5 mL of deionized water and 1 mL K_2_PdCl_4_ solution (containing 0.01 mmol Pd) were put into the round-bottom flask. It was stirring for 5 min at 30°C. Then, 1 mL-solution of NaBH_4_ (0.1 mmol) was injected into the solution and stirring for 5 min. Finally, the resulting precipitation was centrifuged, and washed with H_2_O for 3 times to obtain Pd-Pt/C. Other M-Pt/C was synthesized under the same condition.

#### H_2_ generation upon NH_3_BH_3_ hydrolysis

Generally, H_2_ generation upon NH_3_BH_3_ hydrolysis were carried out at 0°C. 1% Pd-Pt/C and 4 mL of saline solution were added into a 10 mL round-bottom flask (Scheme S1). Under the stirring condition, 1 mL solution of NH_3_BH_3_ (1 mmol of NH_3_BH_3_ is dissolved in saline solution) was added into the flask. The volume of produced H_2_ was determined by measuring the displacement of water in the burette. All the data above are presented as a mean value with an error bar, while each experiment was run three times in parallel.

The turnover frequency (TOF) was calculated as below:

$$\text{TOF}=\frac{V_{H_{2}}}{22.4\times n_{Pt}\times t}$$

Among them, V (H_2_) is the total volume of generated gas, n(Pt) is the moles of Pt, t is reaction time and t = 1 min in this work.

The calculation of H_2_’s yield: Theoretically, 1 mmol of NH_3_BH_3_ is decomposed into 67.2 mL of H_2_ (3 mmol). The yield of H_2_ is the actual amount of H_2_ divided by the theoretical amount of H_2_.

### Quantification and statistical analysis

The error bar is standard error of the mean (SEM), which is the standard deviation of the sample-mean’s estimate of a population mean (It can also be viewed as the standard deviation of the error in the sample mean with respect to the true mean, since the sample mean is an unbiased estimator). SEM is usually estimated by the sample estimate of the population standard deviation (sample standard deviation) divided by the square root of the sample size (assuming statistical independence of the values in the sample):

Equation 2 $${SE}_{\bar{x}}=\frac{s}{\sqrt{n}}$$

In Equation 2: **s** is the sample standard deviation (i.e., the sample-based estimate of the standard deviation of the population), and **n** is the size (number of observations) of the sample.

## Supplemental information

Document S1. Figures S1–S14 and Scheme S1
