# Supplementary material for: Selective and efficient H2 evolution upon NH3BH3 hydrolysis at subzero temperatures
Source: iScience. 2023 Dec 22;27(1):108774. doi: 10.1016/j.isci.2023.108774 (PMC10797192; doi:10.1016/j.isci.2023.108774)
Supplement: Document S1. Figures S1–S14 and Scheme S1 [file mmc1.pdf]

iScience, Volume *27*

Supplemental information

Selective and efficient H_2 evolution upon NH_3BH_3 hydrolysis at subzero temperatures

Qing Zhang, Chen Fang, Yanlan Wang, and Xiang Liu

Supporting Information

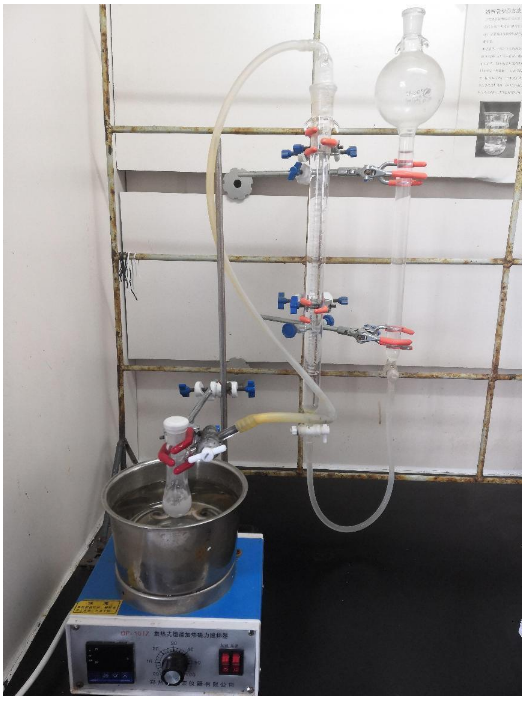

Scheme S1. The experimental setup of NH_3BH_3 hydrolysis. **Related to Figure 6.** The principle of this device is drainage, which is used to record how much gas is produced.

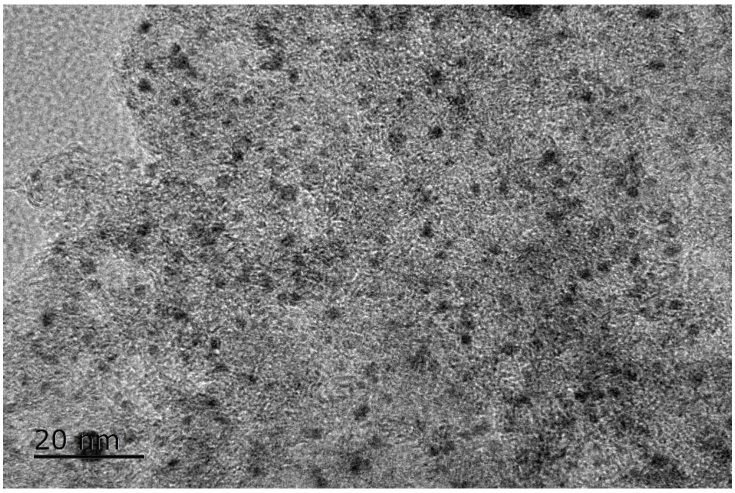

Figure S1. TEM of Pt/C. **Related to Figure 1.** This figure is used to represent the morphology of Pt/C.

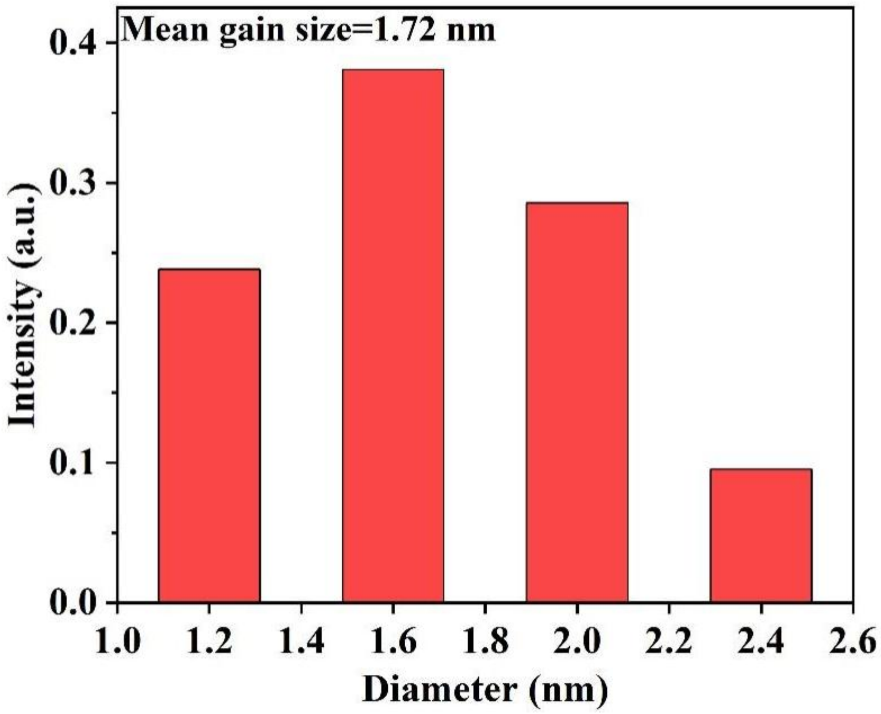

Figure S2. Distribution diagram of Pt/C. **Related to Figure 1 and Figure S1**. This figure is used to represent the mean particle size of Pt/C.

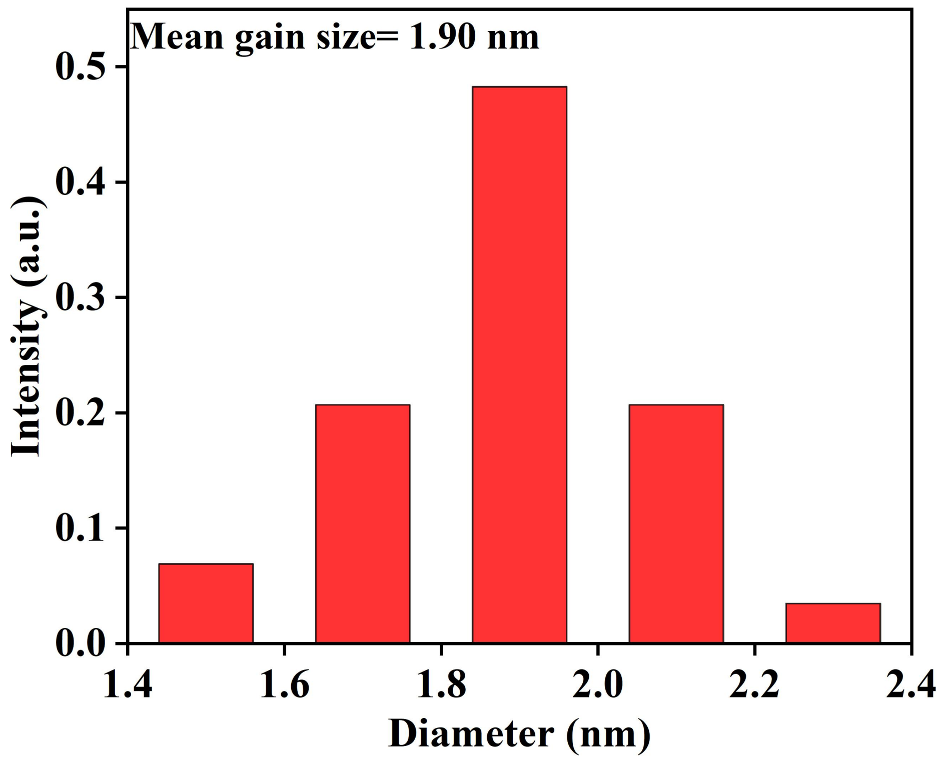

Figure S3. Distribution diagram of Pd-Pt/C. **Related to Figure 1a**. This figure is used to represent the mean particle size of Pd-Pt/C.

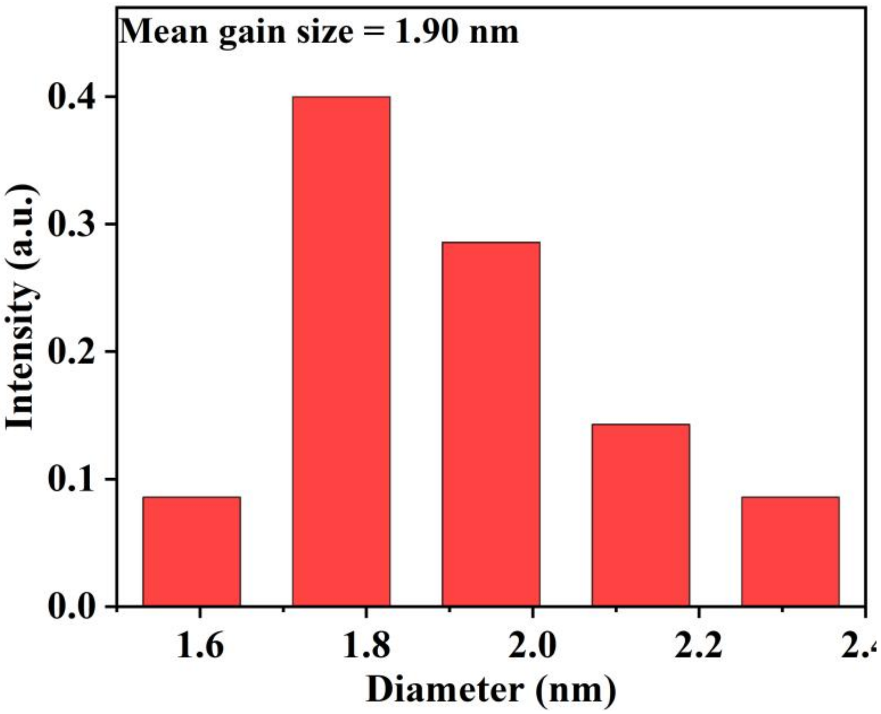

Figure S4. Distribution diagram of Rh-Pt/C. **Related to Figure 1b**. This figure is used to represent the mean particle size of Rh-Pt/C.

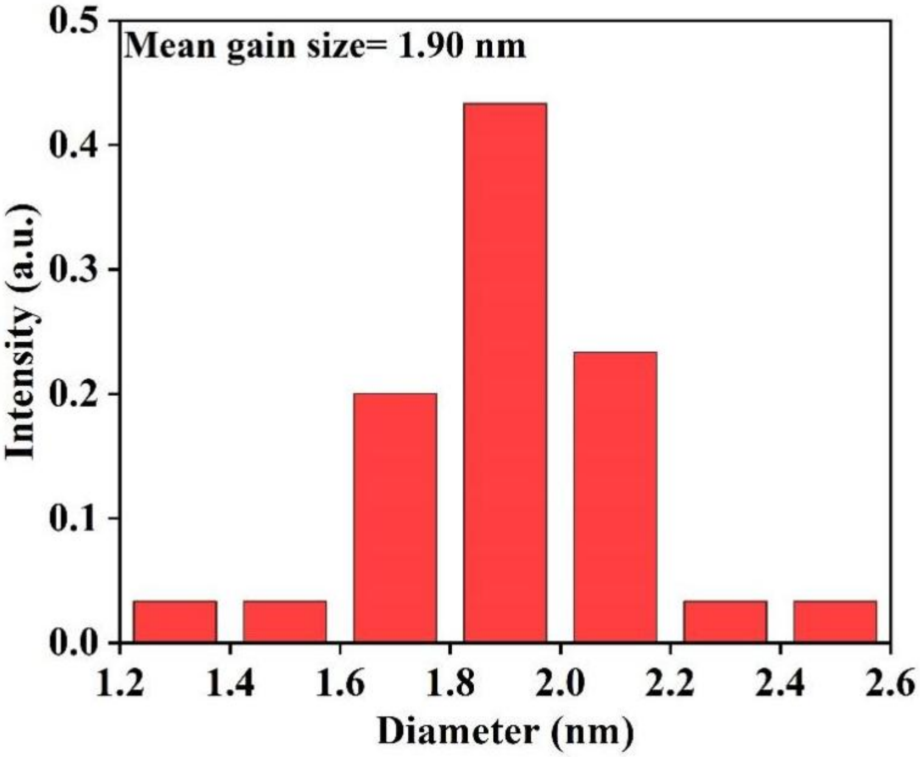

Figure S5. Distribution diagram of Ru-Pt/C. **Related to Figure 1c**. This figure is used to represent the mean particle size of Ru-Pt/C.

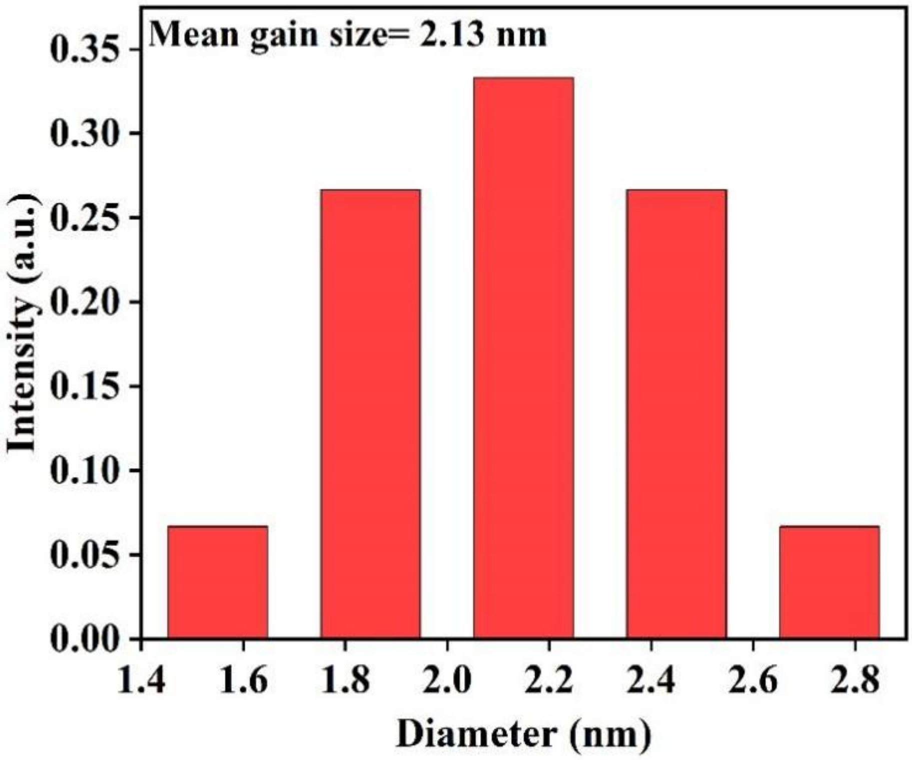

Figure S6. Distribution diagram of Ni-Pt/C. **Related to Figure 1d**. This figure is used to represent the mean particle size of Ni-Pt/C.

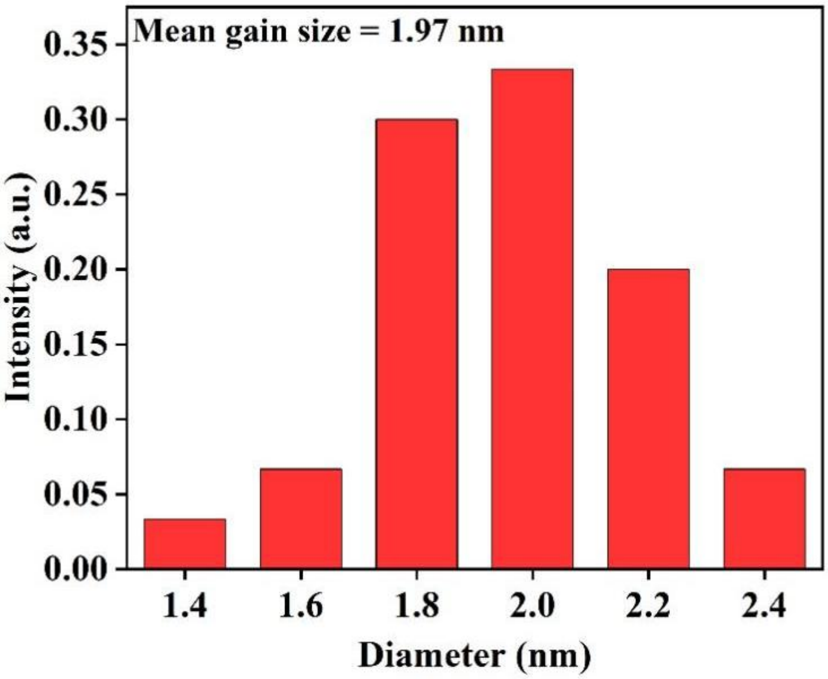

Figure S7. Distribution diagram of Cu-Pt/C. **Related to Figure 1e**. This figure is used to represent the mean particle size of Cu-Pt/C.

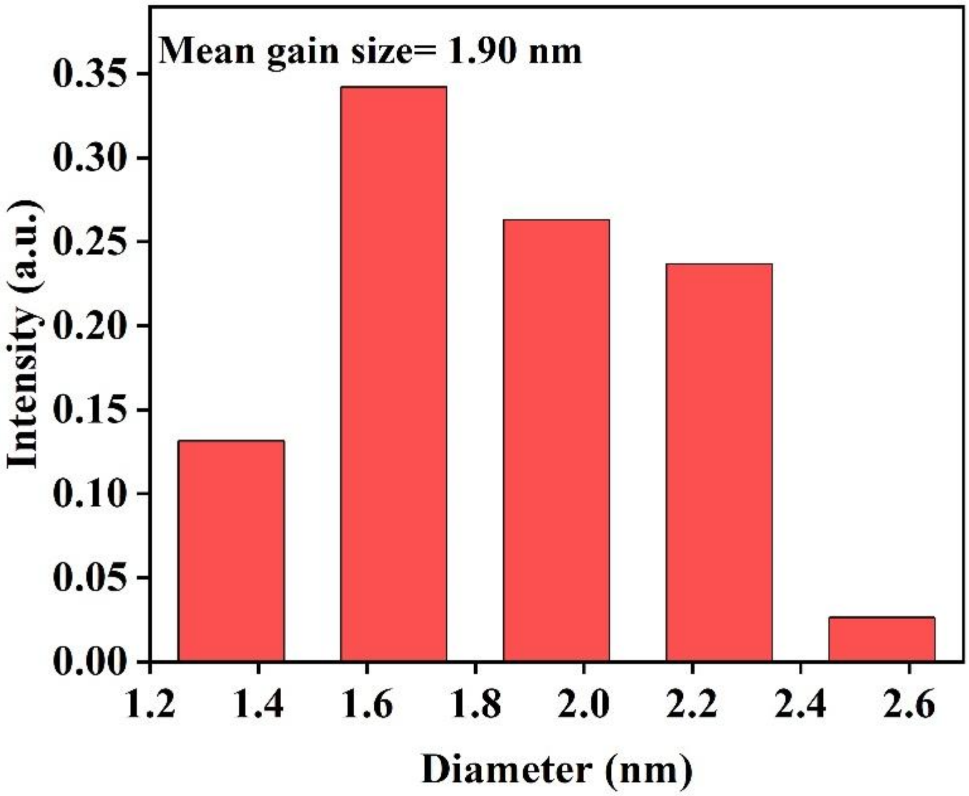

Figure S8. Distribution diagram of Fe-Pt/C. **Related to Figure 1f**. This figure is used to represent the mean particle size of Fe-Pt/C.

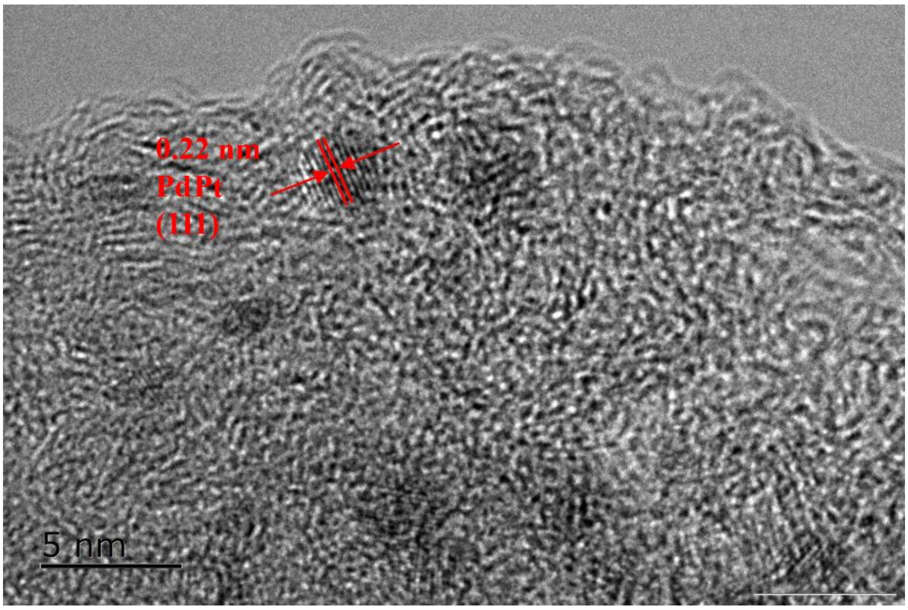

Figure S9. HRTEM of Pd-Pt/C. **Related to Figure 1a**. This figure is used to represent the morphology of Pd-Pt/C.

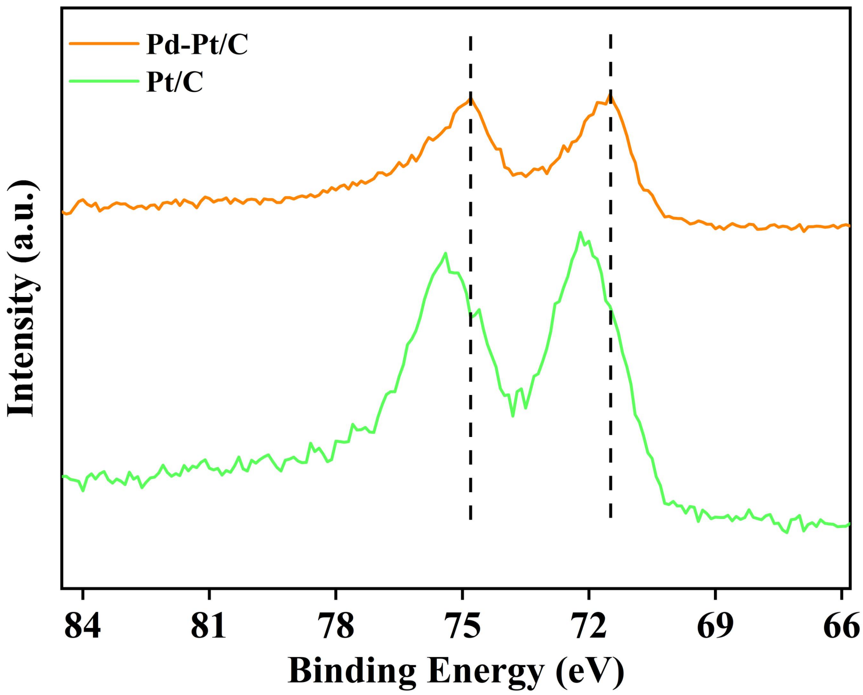

Figure S10. XPS of Pt 4f in the Pd-Pt/C and Pt/C. **Related to Figure 5**. XPS of Pd-Pt/C and Pt/C.

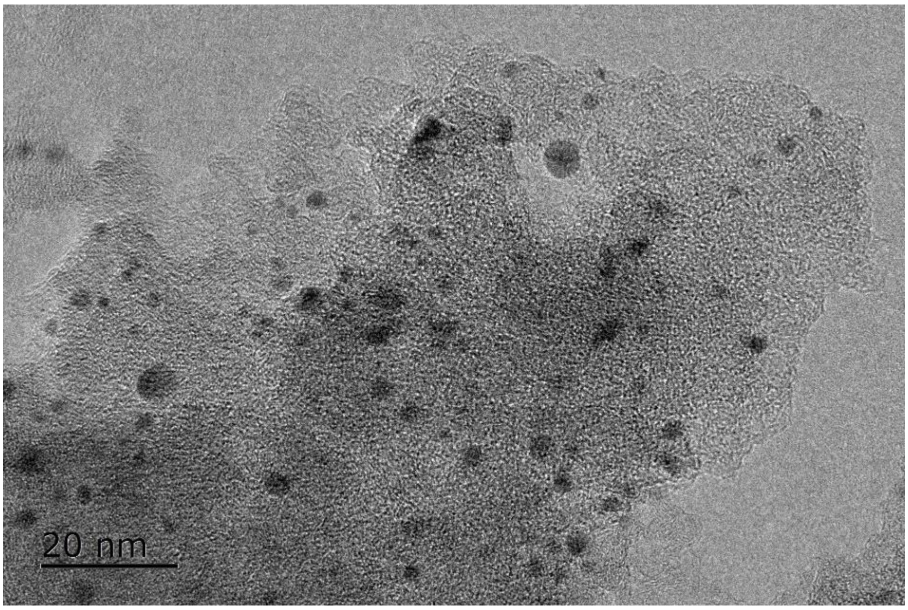

Figure S11. TEM of 5th reused Pd-Pt/C. Related to **Figure 6**. This figure is used to represent the morphology of 5th reused Pd-Pt/C.

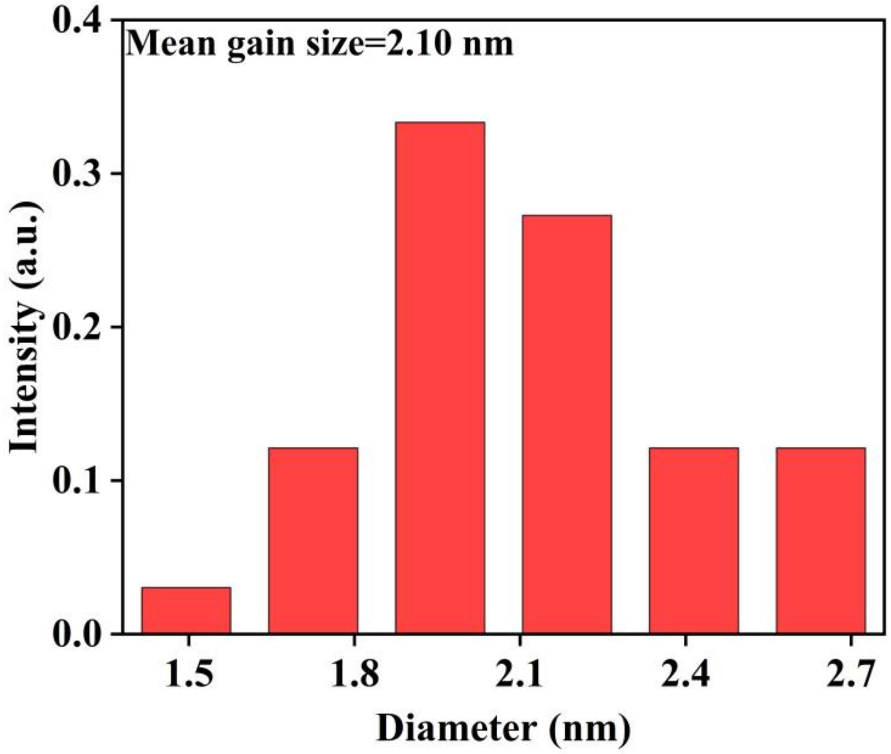

Figure S12. Distribution diagram of 5th reused Pd-Pt/C. Related to **Figure 6 and Figure S11**. This figure is used to represent the mean particle size of 5th reused Pd-Pt/C.

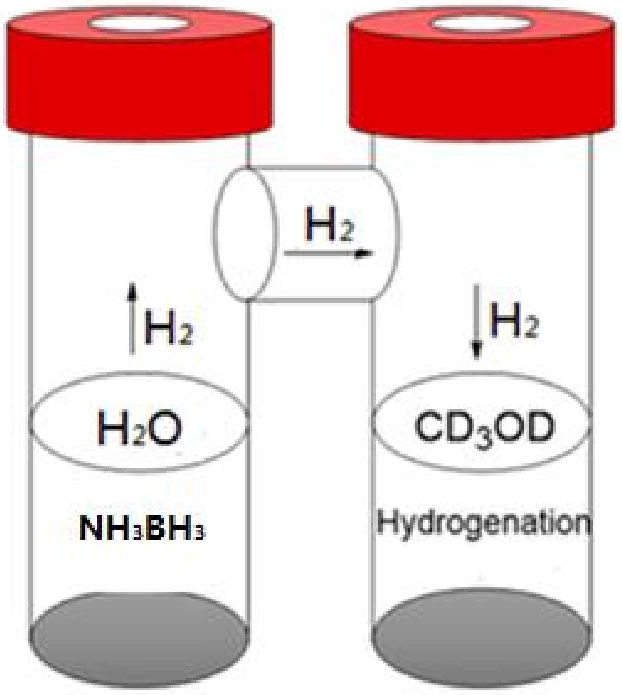

Figure S13. Sealed two-chamber system for norbornene hydrogenation with H_2. **Related to Scheme 3.** Following H_2 evolution. The left tube was used for H_2 generation, and the right one was used for norbornene hydrogenation with generated H_2 in the left tube. The generated H_2 in the left tube transported to the hydrogenation reaction into the right tube through the connecting glass tube.

Hydrogenation of 1,1-diphenylethylene: 50 mg of Pd-Pt/C was added into the left tube. Meanwhile, 10.6 mg (5 mmol % per styrene) Pd/C catalyst was added to the right tube. Air was removed *in vacuo*, and 1.5 mL methanol included 0.1 mmol of norbornene was injected into the right tube. Then 4 mmol NH_3BH_3 dissolved in 5 mL saturated NaCl solution was injected into the left tube. After 5 min, reaction was conducted at 30 °C for 12 h. The combined organic phases were washed with brine (2 × 5mL), dried over anhydrous $MgSO_4$ and concentrated under reduced pressure. The residue was subjected to flash column chromatography with hexanes/EtOAc (20:1) as eluent yielding bicyclo[2.2.1]heptane as light yellow liquid.

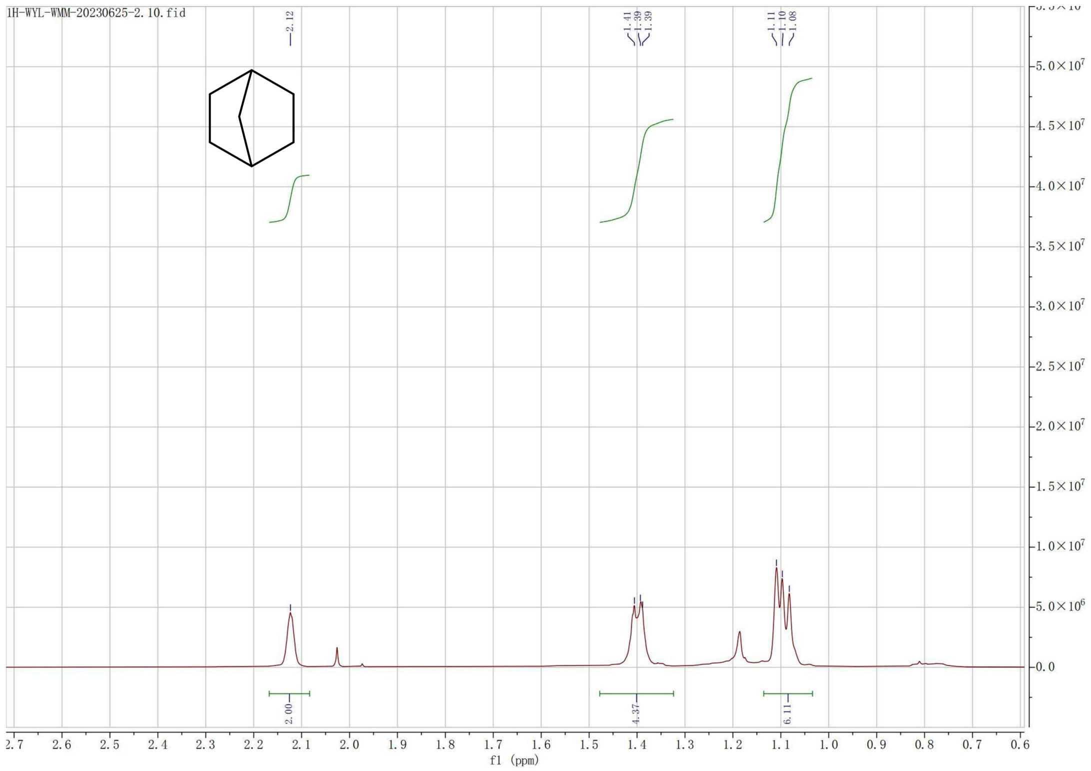

Figure S14. ^{1}H NMR of bicyclo[2.2.1]heptane. Related to **Scheme 3**. ^{1}H NMR (600 MHz, CD_3OD) δ 2.12 (s, 2H), 1.41-1.39 (m, 4H), 1.11-1.08 (m, 6H).
